# Editorial: Recent advances in attempts to improve medication adherence – from basic research to clinical practice, volume II

**DOI:** 10.3389/fphar.2024.1521468

**Published:** 2024-11-25

**Authors:** Przemyslaw Kardas, Ines Potočnjak, Cristina Mihaela Ghiciuc, Maria Teresa Herdeiro, Tamas Agh

**Affiliations:** ^1^ Medication Adherence Research Center, Department of Family Medicine, Medical University of Lodz, Lodz, Poland; ^2^ Sestre Milosrdnice University Hospital Center, School of Medicine Catholic University of Croatia, Zagreb, Croatia; ^3^ Department of Morphofunctional Sciences II – Pharmacology, Clinical Pharmacology and Algesiology, School of Medicine, “Grigore T. Popa” University of Medicine and Pharmacy, Iasi, Romania; ^4^ Saint Mary Emergency Children Hospital, Iasi, Romania; ^5^ Department of Medical Sciences, Institute of Biomedicine (iBiMED), University of Aveiro, Aveiro, Portugal; ^6^ Syreon Research Institute, Budapest, Hungary; ^7^ Medication Adherence Research Group, Center for Health Technology Assessment and Pharmacoeconomic Research, University of Pecs, Pecs, Hungary

**Keywords:** healthcare systems, medication adherence, chronic disease management, digital health technologies, intervention strategies, non-communicable diseases, patient edcuation, polypharmacy

This second volume of the Research Topic, “Recent advances in attempts to improve medication adherence–from basic research to clinical practice,” builds on the previous work of Volume I (Kardas et al., 2023), spotlighting significant new developments in this critical field. Despite decades of focused research, achieving optimal adherence to evidence-based, long-term therapies remains a substantial challenge with direct implications for individual health, public health outcomes, and the sustainability of healthcare systems (Sabaté, 2003). Volume II presents novel insights and innovations aimed at improving adherence from both research and clinical perspectives.

The complexity of non-adherence is influenced by many factors, including the aging global population, the rise of non-communicable diseases, and the prevalence of multimorbidity and polypharmacy (World Health Organization, 2015). Events like the COVID-19 pandemic have further exacerbated these challenges. However, it is increasingly recognized that medication adherence is not solely the patient’s responsibility. It is shaped by a variety of non-patient-related factors and serves as an important indicator of the quality of care. Therefore, rather than assigning blame, all stakeholders must collaborate to create environments that support adherence and enable patients to successfully follow their therapeutic regimens. A recent pan-European survey on medication adherence management emphasized the importance of addressing adherence barriers at multiple levels. It highlighted the need for collaboration among healthcare professionals and advocated for promoting and utilizing digital technologies within clinical practice to improve medication adherence (Kamusheva et al., 2024; Hafez et al., 2024).

To improve adherence, interventions that are proven effective and cost-effective are urgently needed (Ágh et al., 2022). Resources must be used wisely, and this requires rigorous research to support the design, implementation, and benchmarking of interventions. This Research Topic aimed to highlight recent advances in this area by presenting a wide range of studies, from basic theoretical questions to the real-world implementation of interventions, with a focus spanning from individual patients to system-wide approaches.

The theoretical background helps standardize methodologies and benchmark the results of both scientific research and clinical practice. For several years, the ABC consensus terminology and taxonomy set key standards for basic concepts in the field of medication adherence (Vrijens et al., 2012). However, until now, no similar ‘gold standard’ existed for various interventions and technological solutions in this area. The European Network to Advance Best practices and technoLogy on medication adherencE (ENABLE) COST Action (van Boven et al., 2021) has filled this gap, establishing a cohesive set of consensus terms and definitions. These terms include ‘medication adherence technology,’ ‘medication adherence enhancing intervention,’ ‘best practice,’ and ‘reimbursement,’ all of which can help promote consistency in research and clinical practice (Kardas et al.).

To further support benchmarking of national approaches to medication adherence management, ENABLE created a list of key indicators related to medication adherence (IRMAs). The list includes 26 items, covering country characteristics, social/economic factors, therapy- and patient-related factors, condition-related factors, and healthcare system-related factors. An expert survey built a unique database of these indicators for 39 European countries and Israel, providing a solid foundation for tailored interventions aimed at improving adherence and informing health policy development (Ágh et al.).

Medication adherence is most often associated with chronic conditions, but it is equally important in infection treatment, particularly for long-term infections. It is therefore noteworthy that a new instrument was developed to assess medication adherence among tuberculosis (TB) patients in Indonesia (Rianto et al.). The structured questionnaire, validated through rigorous testing, measures socio-economic, healthcare team, and condition-related factors, offering a valuable tool for targeted interventions in TB care.

Available evidence proves that medication adherence in mental conditions is particularly low. A systematic review of current literature put more light over this topic, looking for sociodemographic and clinical predictors of adherence to pharmacological treatment in patients diagnosed with a depressive disorder (Del Pino-Sedeño et al.). Meta-analysis of 39 included studies identified age and ethnicity as key predictors of adherence.

Another group of drugs with particularly low adherence rates is statins, partly due to the asymptomatic nature of hyperlipidaemia, and treatment typically requiring long-term commitment. The recent COVID-19 pandemic created particularly difficult conditions for execution of such therapy. An analysis of nationwide Polish real-word data on prescribing and dispensing allowed for detailed insight into execution of that treatment under unfavourable conditions (Kardas et al.). Although access to statins was maintained, nearly half of the patients remained non-adherent, and approximately 1/7 of prescribed statin doses were never dispensed. These results underscore the need for targeted interventions to improve medication adherence to this drug class of high public health importance.

Even life-threatening conditions like cancer are not immune to the issue of non-adherence. A study from Romania assessed adherence in breast cancer patients receiving oral anti-cancer drugs, finding non-adherence rates between 12.8% and 14.7%, depending on the drug type (Turcu-Stiolica et al.). This highlights the critical need for targeted interventions in cancer treatment.

Identifying non-adherence in patients with cognitive impairments is challenging. Therefore, an important question is whether simple questionnaire tools can be effectively used in this context. A study assessing the diagnostic validity of two self-reported adherence tools - the Morisky Green test and Batalla test - found that these questionnaires effectively identified non-adherence in patients with mild cognitive impairment and dementia, with high specificity but moderate sensitivity (Barnestein-Fonseca et al.). Given their ease of use in clinical practice, the study supports the integration of self-reported methods into routine adherence assessments for cognitively impaired patients, highlighting their practicality.

Achieving adequate adherence is essential for good clinical outcomes. A longitudinal study in children and adolescents with asthma showed that adherence to inhaled corticosteroids, along with correct inhalation technique, was associated with better clinical outcomes such as symptom control, exacerbations, and health-related quality of life (Lizano-Barrantes et al.). However, a third of the participants had suboptimal inhalation technique, pointing to the need for improved patient education. Unfortunately, healthcare professionals often lack sufficient knowledge of proper inhaler techniques and may be inadequately prepared to teach these skills to patients (Maepa et al., 2019).

Under the light of this, somewhat surprising results were provided by a study (Achterbosch et al.), which assessed the effect of shared decision-making (SDM) on medication adherence in patients with chronic obstructive pulmonary disease (COPD) and asthma. Despite a sound theoretical background supporting the use of SDM, this study found no significant association between SDM and adherence. Therefore, there is a room for further investigation into the mechanisms linking SDM and adherence, as SDM alone may not directly influence patients’ adherence to inhalation medications.

On the other hand, in a study examining adherence in patients with severe mental disorders, a psychosocial group intervention focusing on lifestyle behaviours led to a significant improvement in treatment adherence (Sampogna et al.). Interestingly, participants who engaged in moderate physical activity showed better adherence. The study highlights the potential of lifestyle-based group interventions to enhance adherence in mental health settings, suggesting that this approach could be easily integrated into routine clinical practice.

Many interventions targeting adherence are - for various reasons - only short term. This makes the study (Bandiera et al.), which explored the impact of a 6- *versus* 12-month pharmacist-led medication adherence program in patients with diabetic kidney disease, particularly valuable. The findings revealed that patients in the 12-month group had better adherence to antidiabetics and antihypertensive medications compared to those in the 6-month group, indicating that longer intervention durations can lead to sustained improvements in adherence, with potential long-term benefits.

Obvious limitations of such long-term adherence programs are their costs and scarcity of human resources. Novel digital technologies may help to partially address these issue. One example is a study (Larsen et al.) that explored the potential of a mobile app to enhance adherence to selective serotonin reuptake inhibitors (SSRIs) and serotonin-norepinephrine reuptake inhibitors (SNRIs) among antidepressant users. Results showed a noticeable improvement in adherence, with a significant reduction in non-adherence scores following app use. Interestingly, while half of the participants found the app useful, it was not preferred over traditional sources of information.

However, not every simple intervention proves effective. A study evaluating the impact of pocket cards containing key medication information, distributed after a school-based education program, found no significant benefit from the cards. Although the cards did not improve adherence, the structured education program remained valuable, and other tools should be explored to enhance medication knowledge retention (Sakai et al.).

Optimisation of the drug therapy is one of the initial steps toward improving adherence. A study (Jošt et al.) evaluated the effectiveness of pharmacist-led medication reconciliation at the hospital discharge over patient safety and healthcare utilization. Results indicated that medication reconciliation reduced the likelihood of a clinically important medication error by 20-fold. These findings underscore the importance of medication reconciliation as a key measure for patient safety.

The growing need for clinical pharmacy services (CPS) is further underscored by aging populations and the subsequent rise of polypharmacy. Unfortunately, a review of CPS development in Central and Eastern Europe reveals underutilization of pharmacists’ potential to manage medication-related problems (Urbańczyk et al.). Therefore, the authors call for wider adoption of CPS to meet global healthcare challenges effectively.

As polypharmacy becomes an increasingly prevalent problem, negatively affecting medication adherence, effective solutions are more than needed. However, there is only limited information available on their performance in real life settings. This created the background of a cross-sectional study which benchmarked existing polypharmacy management programs in elderly across Europe (Kardas et al.). Findings from over 900 healthcare professionals allowed to assess their effectiveness, applicability, scalability and cost-effectiveness, as well as to create a benchmarking application enabling comparisons across national and European contexts to aid clinicians and policymakers.

An educational intervention aimed at healthcare professionals in primary care demonstrated the effectiveness of training on inhalation technique, as reported in a recent cluster randomized trial (Vázquez-González et al.). The study found that healthcare professionals who received training showed significant improvements in their knowledge and skills regarding inhalation techniques, which are essential for patient adherence and effective asthma management. This highlights the critical role of provider education in achieving optimal patient outcomes and the potential for integrating such interventions into routine clinical practice.

Another study (Bell et al.), adopting a public health perspective to medication adherence, was conducted in Ireland on secondary prevention after stroke. The study revealed ongoing failures to meet adherence targets post-discharge, despite the known importance of medication in recovery. Semi-structured interviews and focus group with healthcare professionals explored the challenges faced by healthcare professionals in community multidisciplinary teams, focusing on continuity of care and its impact on medication adherence. The value of this approach is that it revealed gaps in care organization and communication post-hospitalization.

In conclusion, Volume II of “Recent advances in attempts to improve medication adherence–from basic research to clinical practice” emphasizes the multifaceted nature of adherence challenges. Solutions must go beyond individual efforts and involve a system-level approach that addresses the unique needs of patients. This volume offers promising directions for improving adherence and underscores the importance of collaborative, resource-efficient strategies.
